# AMPDECIDE amputation level patient decision aids: a feasibility study

**DOI:** 10.1186/s12911-025-03084-7

**Published:** 2025-07-01

**Authors:** Alison W. Henderson, Maryam Soltani, Bjoern D. Suckow, Alison R. Kern, Daniel D. Matlock, Joseph M. Czerniecki, Daniel C. Norvell

**Affiliations:** 1 VA Center for Limb Loss and MoBility (CLiMB), 1660 S. Columbian Way, Seattle, WA 98101 USA; 2 VA Puget Sound Health Care System, 1660 S. Columbian Way, Seattle, WA 98101 USA; 3 Department of Vascular Surgery, White River Junction VA Medical Center, 163 Veterans Drive. White River Junction, Hartford, VT 05001 USA; 4 Department of Vascular Surgery, Dartmouth-Hitchcock Medical Center, 1 Medical Center Drive, Lebanon, NH 03756 USA; 5 Departments of Medicine and Geriatrics, University of Colorado, Boulder, USA; 6 VA Eastern Colorado Geriatric Research Education and Clinical Center, Denver, CO USA; 7 Department of Rehabilitation Medicine, University of Washington, 325 Ninth Avenue, Box 359612, Seattle, WA 98104 USA

**Keywords:** Amputation, Shared decision making, Patient decision aids, Feasibility

## Abstract

**Objective:**

This was a feasibility study of the AMPDECIDE amputation level selection patient decision aids (one transmetatarsal vs. transtibial, the other transtibial vs. transfemoral) designed to inform a larger efficacy trial. We intended to gather data about usability of the aids, gather efficacy data about an amputation-level specific knowledge scale, identify any patient-barriers to the use of the decision aids, and evaluate the feasibility of our study methods.

**Design:**

Feasibility study with an uncontrolled before-after design in two medical centers.

**Methods:**

A convenience sample of dysvascular patients (both pre- and post-amputation) seen by either the vascular or orthopaedic surgery services at each facility were recruited. Enrolled patients completed baseline measures (including amputation level knowledge items). They then reviewed the decision aid with a research coordinator, followed by additional measures of control preference, numeracy, literacy and open-ended questions.

**Results:**

Eleven patients were enrolled (9-post amputation, 2 pre-amputation). Patients rated the decision aids as easy to navigate. Nearly all patients expressed a desire to see their personalized mobility and reamputation risks should they be made available. Patients demonstrated 17% improved amputation level knowledge after exposure to the decision aids. In addition, 81% of patients indicated wanting to participate in the amputation level decision. The study encountered difficulties identifying and recruiting patients until greater clinician involvement was included.

**Conclusions:**

The AMPDECIDE patient decision aids and the study measures appear well suited for a larger efficacy trial. Patients were able to digest the information supplied in the aids and responded well to them. The initial recruitment strategy was insufficient; greater clinician involvement may help in the future.

**Clinical trial number:**

Not applicable.

**Trial registration:**

Not applicable.

**Supplementary Information:**

The online version contains supplementary material available at 10.1186/s12911-025-03084-7.

## Background

Shared decision making (SDM) is “the process by which a health care provider communicates information about options, outcomes, probabilities, and scientific uncertainties of available treatment options and the patient communicates his or her values and the relative importance he or she places on benefits and harms.” [1] Patient decision aids (PtDAs) are tools designed to facilitate patient participation in health care decisions by describing the available options and helping patients determine their priorities and values around these options [2–4]. Systematic reviews and meta-analyses have shown that PtDAs improve patient knowledge of the possible options and their associated risks, and reduce decisional conflict, thereby helping patients make decisions that align with their preferences [5–7]. While PtDAs can improve surgical decision making [5], few PtDAs are available to facilitate SDM in vascular surgical conditions [8], especially regarding level of lower extremity amputation.

Patients with advanced dysvascular disease (peripheral artery disease (PAD) and diabetes) may experience disabling pain or non-healing/infected foot wounds often requiring lower extremity amputation. The level along the lower extremity at which an amputation is performed is in part driven by the anatomic extent of disease. However, many other variables factor into the decision at which to amputate. Each amputation level profoundly affects the risks of operative mortality, re-hospitalization, reamputation, functional mobility, and therefore quality of life (QoL) [9–14]. One of the most important factors influencing the amputation level decision is the goal of preservation of mobility, due to its association with QoL [14–17]. More distal amputations generally preserve patient mobility. However, the potential mobility benefits of a more distal amputation may not be realized because of an increased risk of compromised healing of more distal amputations in the setting of advanced PAD [18]. This could necessitate an additional amputation procedure to a higher level, which has been shown to result in a loss of functional mobility that exceeds that which would occur if the amputation was performed at a higher level initially [9–11, 13].

Therefore, PAD patients requiring amputation face competing risks of loss of mobility versus risk of non-healing and reamputation. We developed two novel amputation level PtDAs for patients who require amputation secondary to dysvascular disease to facilitate SDM around the competing risks and benefits (www.ampdecide.org). The objectives of this study were to: (1) evaluate the usability of both PtDAs to facilitate amputation level decision making, (2) examine the validity of our amputation level knowledge scales for a future efficacy trial, (3) identify patient factors that may affect participation in the decision making process, and (4) assess the feasibility of the study procedures in preparation for a larger-scale efficacy trial.

## Methods

### Study design

This was a feasibility study with an uncontrolled before-after design, with two participating centers, one Veterans Affairs (VA) medical center and one university medical center. Study focus was guided by a published feasibility study protocol [19]. 

### Patient decision aids

The two decision aids evaluated inform patients of important evidence-based outcomes (risks of mortality, reamputation and mobility) by amputation level determined from a rapid review of the literature and our qualitative work [20, 21]. Two decision aids are necessary because two separate paired decisions are typically seen in this patient population based on a patient’s medical history and the spectrum of comorbidities. For patients with more distal limb pathology, or less severe underlying pathology, the decision is whether to perform transmetatarsal (TM) or a transtibial (TT) amputation. In cases with more severe pathology and a more distal amputation is not possible, the decision is whether to perform a TT or transfemoral (TF) amputation. The development process of the TM/TT decision aid has been previously published; [21] the development of the TT/TF aid mirrored the same methodological and iterative process following the International Patient Decision Aid Standards (IPDAS), with both aids providing information about amputation level options, as well as patient quote and videos of both patients and providers sharing their experiences and discussing important factors.

### Participants

A convenience sample of patients seen by either the vascular or orthopaedic surgery services at each facility were recruited over a 10-month period. Both pre-operative and post-operative patients were approached for participation. Patients were considered eligible if they were at least 40 years old (to further ensure the reason for amputation was vascular disease), their amputation was a consequence of dysvascular disease, and it was their first amputation mid-foot or higher. Patients were excluded if they had a prior major amputation, if their amputation was due to non-dysvascular etiology, if they had a diagnosis of hemiplegia, quadriplegia, and/or spinal cord injury or significant cognitive impairment and if they had inadequate English language proficiency to consent to participate. Among those recruited pre-amputation, the clinical care team must have previously discussed the possibility of needing an amputation with the patient and amputation surgery was either pending or scheduled.

### Participant baseline and feasibility measures

#### Participants

Data around participant demographic information, amputation level (anticipated level for pre-amputation patients), current mobility, and identification of provider type who informed the patients they needed an amputation were obtained.

#### Usability of PTDAs to facilitate amputation level shared decision making

After interacting with the appropriate PtDA, patients answered semi-structured usability questions derived from our previous qualitative and developmental work (See supplemental materials). Patients were asked if, given the opportunity, they would prefer to see their *personalized* risks for mortality, reamputation, and achieving independent mobility and the reasons for their preferences.

#### Creation and evaluation of amputation level knowledge items

 Amputation level-specific true/false knowledge items were created by the study team members with considerable clinical expertise and were based upon an example content-specific knowledge scale provided by the IPDAS Collaboration [22]. Items consisted of 12 true/false items that directly reference data from the decision aids. To evaluate improvement in knowledge, patients completed the measure prior and after exposure to the appropriate PtDA. Possible scores ranged from 0 to 12, with higher scores indicating greater amputation level knowledge. We added this scale approximately 5 months into the study, so a smaller subset of patients completed this measure.

#### Factors that may influence patient participation in the decision making process

To ensure that patients can effectively engage with the PtDAs, we included a gross cognitive screening measure, the Short Portable Mental Status Questionnaire (SPMSQ) [23], as well as established measures of subjective numeracy and health literacy, where higher scores are associated with greater numeracy and literacy [24, 25]. In addition, we included a measure of control preferences, or how much the patient desires to be involved in medical decision making [26], as well as a specific item about how much they want or would have wanted to be involved in the decision around amputation level selection.

#### Feasibility of study procedures

Study coordinators met with one of the study investigators weekly during active enrollment. Meetings focused on any issues around patient identification, recruitment and enrollment, the study visits and any required follow up, including notable feedback from patients during the study visit that may inform final PtDA modifications and/or future study operational planning. Pertinent data were shared with the study investigative team monthly to ensure that there were no study blocking issues. Key findings from these meetings were organized in an itemized study document for final review.

### Study procedures

At each medical facility, study coordinators regularly communicated with a service line point of contact (nurse manager, physician assistant or surgeon), to assess if there were any eligible patients in clinic or on the ward. Patient eligibility was verified by the study coordinators using the electronic health record. Prior to approaching a potential participant, the study coordinator would confirm with relevant clinical staff which decision aid would be most appropriate (TM vs. TT or TT vs. TF). After consent was obtained, the study coordinator administered the pre-PtDA exposure measures (baseline demographic information and amputation knowledge items). Then, the coordinator oriented the patient to the overall purpose of the research and the online version of the PtDA. The patient was allowed to maneuver through the online PtDA, being encouraged to verbalize their experience via a “think aloud” methodology to identify thought processes and behavior patterns, as well as any issues with the tool [27]. Think aloud interview notes were organized into page-by-page summaries for evaluation by the larger research team. After the patient navigated through the aid, post-exposure assessments (amputation knowledge, numeracy and literacy) were collected.

### Data analysis

Data from both sites were combined for analysis. Descriptive statistics were computed (frequencies and means). For the knowledge items, change scores were created (pre- and post-PtDA exposure), and frequencies of change scores were computed. All quantitative analyses were run using Stata v.16 (StataCorp. 2019. Stata Statistical Software: Release 16. College Station, TX: StataCorp LLC). Open-ended responses and “think aloud” feedback collected by study coordinators were consolidated, organized and summarized by one study coordinator and discussed among the investigative team to determine changes to be made.

## Results

### Participants

Eleven patients were enrolled (9-post amputation, 2 pre-amputation). Participants were predominantly married, white male veterans. The average age of participants was 70, with a majority (67%) having received a TM amputation. Participants’ current mobility levels varied widely, ranging from primarily bedridden to unlimited community ambulators. And while 45% of participants learned they needed an amputation from a vascular surgeon, others learned of this need from a variety of providers (Table 1).

**Table 1:** Baseline factors for enrolled patients

Variable	*N* (%) or M (SD), range
**Demographic factors**	
Age	70.4 (9.81), 57–90
Male	11 (100%)
Marital Status (*n* = 10)	
Married	5 (50%)
Single (never married)	2 (20%)
Divorced	3 (30%)
Race	
White	10 (91%)
Black	1 (9%)
Not Hispanic or Latinx	11 (100%)
Enrolled at VA Medical Center	8 (73%)
Living situation	
At home alone	6 (55%)
At home with another	4 (36%)
Nursing home	1 (9%)
Self-rated Health	
Fair	4 (36%)
Good	4 (36%)
Very good	3 (27%)
**Amputation factors**	
Patient group	
Pre-op TM/TT	0 (0%)
Post-op TM/TT	6 (55%)
Pre-op TT/TF	2 (18%)
Post-op TT/TF	3 (27%)
Known amputation level (*n* = 9)	
TM	6 (67%)
TT	1(11%)
TF	2 (22%)
Anticipated amputation level (*n* = 2)	
TM	0 (0%)
TT	1 (50%)
TF	1 (50%)
Provider who first told patient they needed amputation	
PCP	1 (9%)
Podiatrist	2 (18%)
Vascular surgeon	5 (45%)
Orthopaedic surgeon	1 (9%)
Other	2 (18%)

Note: Transmetatarsal = TM; Transtibial = TT; Transfemoral = TF; Primary Care physician = PCP

### Usability of the PTDAs to facilitate amputation level shared decision making

Participants generally had favorable responses to the PtDAs. All found the aids easy or very easy to navigate. When asked about their own specific risks, nearly all participants wanted to know their individual risk of mortality, reamputation and chance of achieving independent mobility (Table 2).

**Table 2:** Usability of the decision aid

Variable	*N* (%)
How easy was it to maneuver through the aid?	
Easy	2 (33%)
Very easy	4 (67%)
Was it hard to learn about mortality risk?	
Yes	2 (33%)
No	4 (67%)
Is it important to learn about mortality risk?	
Yes	6 (100%)
Of the outcomes presented, which is the most important to learn about?	
Dying	1 (17%)
Healing	2 (33%)
Walking	3 (50%)
Are patient-specific risks in a decision aid a good idea?	
Unsure	2 (33%)
Good idea	2 (33%)
Very good idea	2 (33%)
Would you want to know your personal mortality risk?	
Yes	6 (100%)
Would you want to know your personal healing risk?	
Yes	5 (83%)
Would you want to know your personal mobility risk?	
Yes	6 (100%)

Summarizing the “think aloud” notes, many patients were surprised that there was a decision to be made about amputation level (a pre-amputation patient stated “I don’t understand when you talk about more surgery options [referencing reamputation]. I don’t understand how I can have additional surgery on this leg”). Many post-amputation patients indicated that they weren’t exposed to this information prior to their amputation (“If I had seen this prior to surgery, it would have given me a little more info and I would be asking about death, lifestyle, independence, and self-identity. It explained it better than doctors. The doctors didn’t discuss this in depth”). Finally, several patients noted that they wanted to view the tool with their family and loved ones, and others with their care provider. There were few proposed changes, but two participants indicated they believed one of the videos was biased toward a certain amputation level.

### Evaluation of amputation level knowledge items

While only a subset of patients completed the pre/post knowledge items (7 of the enrolled 11 patients), patient amputation level knowledge appeared to increase. Of the 5 participants exposed to the TM/TT PtDA (all post-amputation), 3 of the 5 individuals demonstrated increased knowledge after exposure to the aid (2 showed no change) for an overall average increase of 2 points (17% increase). Of the 2 participants exposed to the TT/TF PtDA (both post-amputation), 1 of the 2 individuals demonstrated increased knowledge after exposure to the aid (the other showed no change), for an overall average increase of 1.5 points (12% increase).

### Factors that may influence patient participation in the decision making process

Regarding the brief cognitive screening measure, study participants appeared cognitively intact with all scoring a 10 out of 10 on the SPMSQ. Similarly, they generally scored high on both subjective numeracy and health literacy, indicating that all were sufficiently able to engage in the PtDA and interview questions. Most patients indicated wanting to participate in general medical decisions, with 54% indicating they would prefer to make the final decision and 27% indicating they would prefer to share in the decision with their provider. This preference was mirrored in patient preferences around amputation level selection (Table 3).

**Table 3:** Factors that may influence patient participation

Variable	*N* (%) or M (SD), range
**Control Preferences**	
Control preferences, general	
Prefer to make final decision	1 (9%)
Prefer to make final decision after considering MD opinion	5 (45%)
Share responsibility with MD	3 (27%)
Prefer MD make final decision after considering my opinion	2 (18%)
Control preference, amputation level selection	
Prefer to make final decision	2 (18%)
Prefer to make final decision after considering MD opinion	4 (36%)
Share responsibility with MD	3 (27%)
Prefer MD make final decision after considering my opinion	2 (18%)
**Numeracy**,** Health Literacy and Cognition**	
Numeracy total (*n* = 9)	4.5 (0.82), 3-5.9
Numeracy ability	4.75 (0.94), 3.75-6
Numeracy preference	4.25 (1.45), 1.75–5.75
Health literacy -- How often someone helps with reading materials (*n* = 9)	
Often	1 (11%)
Sometimes	1 (11%)
Occasionally	1 (11%)
Never	6 (67%)
Health literacy—how often difficulty understanding writing about health	
Some of the time	3 (33%)
A little of the time	1 (11%)
None of the time	5 (56%)
Health literacy—how confident filling out forms	
All the time	8 (89%)
Most of the time	1 (11%)
SPMSQ (*n* = 7)	10 (0) 10–10

### Feasibility of study procedures

We intended to enroll up to 36 participants across two sites (a Veterans Affairs Medical Center and a University Medical Center). As noted above, we were only able to enroll 11 participants out of a possible 18 identified, less than one-third of our anticipated goal. There were two noted barriers to enrolling patients. The first was consistent patient identification. Because patients facing a dysvascular amputation may be seen when amputation is more or less imminent, they may be seen in a variety of settings (in the emergency department (ED), wound care clinic, etc.). In addition, providers who perform amputations may vary between facilities, making it challenging to engage the appropriate provider. For example, at the VA medical center, amputations were performed by podiatric, vascular, general and orthopedic surgeons, each with its own service line. At the university medical center, vascular surgeons performed all amputations. It should also be noted that potential participants needed to be identified by a clinician as a candidate to view two different amputation level options. It may have been that limited clinician awareness about the value of shared decision making precluded patient identification. Future studies will provide greater clinician education about the value of engaging patients in the decision making process, even if there is an option that is more preferred over another. In addition, patients were often identified very close to their amputation procedure, which inhibited an opportunity for research staff to connect with the prospective participants. Identifying patients when they are farther out from the amputation procedure (i.e., during outpatient wound care clinic visits) might be more successful to identify and engage patients.

Once a patient was identified as an appropriate potential participant, study coordinators encountered an additional barrier. At the university hospital, study coordinators are welcome to approach patients to participate in research studies without a “warm handoff” from a clinical provider (e.g., when a clinical provider speaks to the patient and asks if they would be interested in hearing a study coordinator describe the study and determine if the patient would like to participate). Without this clinician-mediated interaction, enrollment at the university hospital was lower until recruitment protocol was modified to include greater clinician involvement (9 patients were approached and 3 of the 9 participated). In contrast, at the VA medical center, where clinician-mediated interactions are required by the institutional review board, 9 patients were approached and 8 of the 9 participated. At the end of study, final modifications were made to the aids in response to patient feedback (e.g., re-recording a video that was identified as biased).

## Conclusion

Feasibility studies play an important role in planning and preparation for a larger trial of an intervention or of the implementation of an intervention [28]. Such studies allow for the identification of areas of uncertainty or proposed methods that require modification, as well as to test preliminary intervention effects [29]. The completion of a feasibility trial allows for a more successful implementation of a larger scale trial, maximizing resources available, although feasibility trials are often under-reported, particularly in surgical settings [30]. The current feasibility trial evaluated the usability of two decision aids, a novel amputation knowledge measure, patient characteristics that might influence participation in SDM and our study procedures, all in preparation for a larger efficacy trial. We found that the patients found the tools easy to navigate and that they would likely provide additional important knowledge for patients facing amputation level decisions. We also noted opportunities to improved study procedures for a future trial including formal education to providers on shared decision making around amputation level choices.

As efforts to integrate SDM into medical care expand, the use of patient decision aids has also increased. However, not all decision aids are effective, and although many tout the benefits of decision aids, even among well-tested aids, their clinical use remains low [31]. A 2019 study of 108 decision aids that had completed randomized controlled trials of their effectiveness, only 21.4% were implemented into clinical practice after conclusion of the trial [32]. Reasons for lack of uptake were a lack of implementation plan, having a decision aid that was difficult to update (notably, aids that were not online), aids not specifically designed with clinical use in mind, and clinic-related barriers (clinicians not knowing about the decision aid, not knowing about the benefits of decision aids, and not having time to use or share the aid) [32]. 

A recent trend to overcome barriers to the use of PtDAs is by engaging patients through online, mobile, and digital routes including website portals, electronic health records, and secure messaging [33]. A systematic review identified six randomized trials that assessed the impact of electronic PtDAs on SDM and patient-centered outcomes in patients with serious illnesses. All but one study demonstrated improvement in at least one patient-centered outcome [34]. Furthermore, the report concluded that electronic PtDAs may reduce unnecessary treatment in comparison with informational pamphlets [34]. PtDAs integrated into a clinical workflow (i.e., available electronically and accessible via the electronic health record) may allow for improved documentation of SDM and save busy clinicians time within the clinical encounter [35], particularly at the point of care [36]. Clinician education around the value of shared decision making will also facilitate use of these tools [37]. 

Although a small sample, and with most patients viewing the aid post-amputation and outside a clinical encounter, participant knowledge still improved by 17%, consistent with other published PtDA interventions [38], and greater improvement than a PtDA for vascular surgery [39]. We anticipate that in an efficacy trial, where patients would be viewing the aids prior to amputation and within the context amputation level decision making, the improvement in amputation level knowledge may be even greater. The items created do appear able to demonstrate sufficient clinical change to be used in a future trial.

Patients enrolled in the study appeared to have the cognitive capacity, numeracy and literacy skills to understand the data presented in the decision aids (including the average percentages presented for risks of reamputation and changes of restoration of mobility). While there is justifiable concern about how data are presented to patients, both in complexity and to prevent bias [40, 41], the numeric data presented in this PtDA appears to be well-received and comprehended by patients. Similarly, patients appeared to have sufficient health literacy to comprehend the data presented. The text of both aids was guided by IPDAS standards, which recommend writing at an 8th grade level or below [42]. The addition of provider and patient videos may serve as a compliment for individuals with low health literacy, along with providing greater patient engagement with the tool [43]. 

Consistent with other studies, patients indicated a desire to participate in their medical decisions, and in decisions around amputation level specifically [44]. While it is noted that some dysvascular amputation level decision may be dictated fully by extent of disease, thus preventing clinicians from engaging in SDM [45], patients may still want to be involved in the decision making process.

Patient identification and recruitment proved to be a challenge in this feasibility trial. It may be that in the effort to cast a wide net, enrolling both pre-amputation and post-amputation patients, study coordinators and clinical staff were unable to sufficiently focus their energy recruiting. While it may be more opportune to identify patients sufficiently upstream from amputation (e.g., in diabetes or wound care clinics), there will likely always be emergent cases when a patient is admitted through the emergency department (ED) and needs an amputation imminently; therefore, providing an option for patient engagement in this setting remains important, even if challenging. A recent review of SDM in surgery noted that none of the 74 published studies in the review included SDM in an emergent setting [46]. The authors noted that most emergencies do not preclude an opportunity for discussion [47], even if the options are between surgery and death. Rather than disregard enrolling challenging patient groups, we propose identifying a “champion” within each service line, a clinician who understands and supports the value of the intervention and takes some ownership of appropriate patient identification. Similarly, identifying a champion within the ED can facilitate patient identification in that fast-paced environment [48]. 

Several limitations to this study should be noted. First, the patient sample was all male, and overwhelmingly post-amputation, and primarily Veterans. As such, generalizations to more varied populations should be carefully considered. Similarly, while development of the aids included ensuring an 8th grade literacy level, these tools may not be acceptable to individuals with lower health literacy or subjective numeracy. In addition, many of the participants were post-amputation (and with a more distal amputation), which may provide a different vantage point when viewing the tool than a pre-amputation patient facing the decision between a TT and TF amputation. The larger efficacy study will focus exclusively on a pre-amputation population and recruiting more females and people of color, addressing the current limitation. The efficacy study is intended to be implemented in a larger number of medical facilities, which will provide greater opportunities to address implementation of study procedures in different settings with different practice cultures.

The data from this feasibility study will inform an efficacy trial of the use of these tools in amputation level selection. Beyond the immediate larger trial, future studies may examine the efficacy of *personalized* amputation level decision aids. With personalized data, patients will be more likely to weigh the evidence and balance the risks of available option to ensure that their choice of level is congruent with their preferences and priorities [40]. 

## Electronic supplementary material

Below is the link to the electronic supplementary material.

Supplementary Material 1

## Data Availability

The datasets generated and/or analysed during the current study are not publicly available due VA data security requirements but may be available from the corresponding author on reasonable request.

## Abbreviations

ED: Emergency Department
PtDA: Patient Decision Aid
PAD: Peripheral Artery Disease
QofL: Quality of Life
SDM: Shared Decision Making
SPMSQ: Short Portable Mental Status Questionnaire
TF: Transfemoral
TM: Transmetatarsal
TT: Transtibial
VA: Veterans Affairs
