# Supplementary material for: AMPDECIDE amputation level patient decision aids: a feasibility study
Source: BMC Med Inform Decis Mak. 2025 Jul 1;25:218. doi: 10.1186/s12911-025-03084-7 (PMC12210496; doi:10.1186/s12911-025-03084-7)
Supplement: Supplementary file 1 — Supplementary Material 1 [file 12911_2025_3084_MOESM1_ESM.docx]

Supplemental material (Usability interview guide)

| **Patient Decision Aid Usability Questions** |
| --- |

- - - 1. First, I would like to better understand your experience of how your amputation level was determined
         1. Did you feel you had a choice around amputation level (below knee vs above knee)?

|  |
| --- |

- - - - 1. Can you describe how your amputation decision made?

|  |
| --- |

- - - - 1. Who helped you with making your decision?

|  |
| --- |

- - - - 1. Were you shown any educational tools or decision aids to help you in the decision-making process? If so, what were they? How did you feel about them? Did they help you make your decision?

|  |
| --- |

- - - - 1. How did you feel about the decision-making process?

|  |
| --- |

- - - 1. Now, I’m going to ask you some questions about the decision aid. What is your reaction in general to this decision aid?

|  |
| --- |

- - - - 1. Would this tool be helpful for patients who might need an amputation?

| Yes | No |
| --- | --- |
| 1□ | 0□ |

[If no] What would you change? [Videos/text/layout/photos]?

|  |
| --- |

- - - 1. How easy or hard was this decision aid to understand?

|  |
| --- |

- - - - 1. Was there anything you didn’t understand? What and why? {are there words you don’t know/understand?

|  |
| --- |

- - - 1. Some people have told us they learned something new from the decision aid, while others have not. What about you?

|  |
| --- |

- - - 1. We have included some sensitive topics in this aid, including the disease processes that resulted in the amputation and how it can affect the risk of dying in the year after amputation. What do you think about this page? Would you want this information included?

|  |
| --- |

- 1. Would you want to know about your personal 1-year risk of dying, given your specific health status? Why or why not?

|  |
| --- |

- - - 1. Would you want to know your personal chance of achieving a certain level of mobility at one-year post-amputation? Your personal chance of needing an additional amputation on the same leg? Why or why not?

|  |
| --- |

- - - 1. What are your thoughts about the page that discusses concerns about independence and living situation? How has your experience been similar or different from what was described on the aid?

|  |
| --- |

a. What on this page would you change/add/remove?

|  |
| --- |

- - - 1. What do you think of the visuals (images, videos) included in the decision aid?

|  |
| --- |

- - - 1. What advice would you give us to improve it?

|  |
| --- |

- - - 1. What questions do you have after seeing this decision aid?

|  |
| --- |

- - - 1. Is there something important that individuals who need an amputation should know about that isn’t in the decision aid?

|  |
| --- |

- - - 1. Some people find that a decision aid may help them figure out what is important to them, that is, what they value. Can you tell me if this decision aid would have helped you to decide what was important to you? If so, how? (Please give examples)

|  |
| --- |

- - - 1. What would you think if your doctor gave or referred you to this decision aid?

|  |
| --- |

- - - 1. Would seeing this decision aid prior to your amputation have changed the way you discuss things with your doctor?

|  |
| --- |

Your friends/family/caregiver?

|  |
| --- |
